# Shiga Toxin Subtypes, Serogroups, Phylogroups, RAPD Genotypic Diversity, and Select Virulence Markers of Shiga-Toxigenic *Escherichia coli* Strains from Goats in Mid-Atlantic US

**DOI:** 10.3390/microorganisms10091842

**Published:** 2022-09-15

**Authors:** Eunice Ndegwa, Dahlia O’Brien, Kwame Matthew, Zhenping Wang, Jimin Kim

**Affiliations:** 1 Agricultural Research Station, Virginia State University, Petersburg, VA 23806, USA; 2 Virginia Cooperative Extension, Virginia State University, Petersburg, VA 23806, USA; 3 College of Agriculture and Related Sciences, Delaware State University, Dover, DE 19901, USA; 4 Columbia College, Columbia University, New York, NY 10027, USA

**Keywords:** STEC, Shiga toxin subtypes, goats, serogroups, phylogroups, virulence genes

## Abstract

Understanding Shiga toxin subtypes in *E. coli* from reservoir hosts may give insight into their significance as human pathogens. The data also serve as an epidemiological tool for source tracking. We characterized Shiga toxin subtypes in 491 goat *E. coli* isolates (STEC) from the mid-Atlantic US region (*stx1* = 278, *stx2* = 213, and *stx1*/*stx2* = 95). Their serogroups, phylogroups, M13RAPD genotypes, *eae* (intimin), and *hly* (hemolysin) genes were also evaluated. STEC-positive for *stx1* harbored *Stx1c* (79%), *stx1a* (21%), and *stx a*/*c* (4%). Those positive for *Stx2* harbored *stx2a* (55%) and *Stx2b* (32%), while *stx2a/stx2d* and *stx2a/stx2b* were each 2%. Among the 343 STEC that were serogrouped, 46% (n = 158) belonged to O8, 20% (n = 67) to 076, 12% (n = 42) to O91, 5% (n = 17) to O5, and 5% (n = 18) to O26. Less than 5% belonged to O78, O87, O146, and O103. The *hly* and *eae* genes were detected in 48% and 14% of STEC, respectively. Most belonged to phylogroup B1 (73%), followed by D (10%), E (8%), A (4%), B2 (4%), and F (1%). M13RAPD genotyping revealed clonality of 091, O5, O87, O103, and O78 but higher diversity in the O8, O76, and O26 serogroups. These results indicate goat STEC belonged to important non-O157 STEC serogroups, were genomically diverse, and harbored Shiga toxin subtypes associated with severe human disease.

## 1. Introduction

*E. coli* is a common inhabitant of the mammalian gut either as a commensal, an opportunistic pathogen, or a primary pathogen, causing disease in animals and humans [1]. STEC, both O157 and non-O157 STEC, are commonly found in the gut of healthy ruminants and are considered the major reservoir hosts for the strains pathogenic to humans [2,3,4]. These bacteria are transmitted to humans by direct animal contact or by ingestion of food or water that has been contaminated with animal fecal material. STEC cause diarrhea, hemorrhagic colitis, and hemolytic-uremic syndrome (HUS) in humans worldwide but are also found in healthy individuals.

Shiga toxins are broadly classified into two types: Shiga toxin1 (*stx1*) and Shiga toxin 2 (*stx2*). Several subtypes of both Shiga toxin 1 and 2 are known to exist and STEC may contain one, both, or a combination of subtypes [5,6]. A recently developed nomenclature for Shiga toxins includes *stx1* variants *Stx1a*, *Stx1c*, and *Stx1d*, while *stx2* includes *Stx2a*, *Stx2b*, *Stx2c*, *Stx2d*, *Stx2e*, *Stx2f*, and *Stx2g* [7].The importance of characterization and typing of the Shiga toxins is underscored by the fact that some Shiga toxin types and variants are correlated more closely with severity of disease in humans than others. In most studies, infection with STEC harboring the *stx2a* and *stx2d* genotypes are associated with severe disease in both human and in vitro models compared with *stx1* and some other variants of *stx2* reviewed in [8,9,10,11,12]. For example, *stx2a* and *stx2d* purified toxins were found to be twenty-five times more potent in VERO monkey kidney and primary human renal proximal tubule epithelial cells than *stx2b* and *stx2c*. In the same experiment, *stx2b* and *stx2c* had similar potency to *stx1* in vivo in mice, while *stx2a* and *stx2d* had from 40 to 100 times potency [10]. As a result of development of the recent PCR subtyping method and its application in STEC clinical isolates, it was also observed that STEC possessing variants of the *Stx2* (*stx2a*) had been more commonly associated with severe disease in humans than STEC possessing other *stx2* variants (*stx2e*, *stx2f*, and *stx2g*) which were of lower virulence [7,8,13].Furthermore, certain Shiga toxin subtypes are associated with specific species of animal reservoirs [8] and rate of shedding from the host, and can, therefore, serve to track the source of the STEC.

The diversity of Shiga toxin subtypes in ruminants, the key STEC reservoirs, have not been widely studied. Although severe STEC infections in humans have in the past been known to be predominantly due to O157 serogroups, recent studies have underscored the importance of non-O157 in human disease in the US [14,15] and globally [16,17,18,19]. Understanding the different Shiga toxin subtypes in all STEC from ruminants and characterizing the serogroups to which they belong will elucidate their significance as animal and public health pathogens. Similarly, genotyping of STEC is a useful tool to describe the clonal relatedness of the different isolates and identify any unique genetic patterns based on serogroups or virulence gene combinations, if any. The information is relevant for epidemiology studies, especially source tracking during disease outbreaks. Several methods exist for genotyping bacteria, including pulsed field gel electrophoresis (PFGE), multilocus sequencing technique (MLST), random amplified polymorphic DNA PCR (RAPD), and, recently, whole genome sequencing (WGS) techniques. The applications, advantages, and disadvantages of each of these methods as genotyping tools has been extensively reviewed and described [20]. Due to the reproducibility, cost effectiveness, speed, and practicality, the RAPD method genotyping, especially when dealing with many isolates, has been described as a great tool for evaluating clonal diversity of *E.coli* isolates [21,22,23] and for fingerprinting of *E. coli* O157 strains [24,25,26]. To understand the risk that the various ruminant species (as reservoirs of STEC) pose to human health, more epidemiological studies are needed to determine the prevalence and diversity of the STEC subtype in each reservoir species in the different geographic regions. Thus, in this study, we characterized the Shiga toxin subtypes, corresponding serogroups, select virulence markers, M13 RAPD genotypes, and phylogroups of STEC isolated from goats in the mid-Atlantic US.

## 2. Materials and Methods

### 2.1. STEC Isolates

The STEC evaluated in this study were from fecal samples collected from research animals at Virginia State University (VSU), Delaware State University, and from select producer farms in Virginia and Delaware between 2017 and 2020. The samples were collected from goats ranging from 3 weeks old to adults over 6 years old. Isolation of *E. coli* from fecal samples was done by overnight enrichment of 200 mg fecal sample in 2 mL of Tryptic Soy Broth (TSB) broth. The broth was serially diluted and 10^5^ and 10^6^ dilutions plated on EMB agar. Three to four colonies with metallic sheen were picked from the highest dilution with 30–100 colonies. All *E. coli* isolates were cultured in Luria broth and stored in 20% glycerol at −80 °C until further processing. Some of the STEC used in this study were from a previous study at VSU and these were revived from frozen isolates by subculturing twice in Luria broth.

### 2.2. DNA Extraction

Each STEC isolate was cultured overnight in Luria broth. The broth was used for DNA extraction following a simple boiling method as previously described [27] with a few modifications. In brief, two milliliters (2 mls) of overnight Luria broth containing the isolates was centrifuged at 14,800 rpm (full speed) for 3 min at room temperature. The supernatant was poured out and the pellet further re-suspended in one milliliter (1 mL) of molecular grade water by vortexing. The bacteria suspension was further centrifuged at full speed for 3 min. The supernatant was poured out and the pellet re-suspended by vortexing in 200–500 µL molecular grade water. The suspension was boiled for 5 min at 100 °C using a tabletop heating block to lyse the bacteria. The suspension was centrifuged again at full speed for 4 min to pellet the bacteria lysate, and 150–300 µL of the supernatant containing the DNA was transferred to a new tube. DNA concentration and purity was measured using a Nanodrop 2000 c, and samples were stored at −20 °C until further processing.

### 2.3. Evaluation of Shiga Toxin Subtypes in STEC

Isolates were screened for Shiga toxins using the primers previously described [7]. For all the STEC isolates, a recently developed simple PCR Shiga toxin subtyping method was used. Primers used included *stx1* and *stx2* detection and subtyping primers described in the paper [7] (Table 1). Two hundred (200 ng) of DNA, 0.5 μL of 10 uM primer, 12.5 μL of the Amplitaq Gold master mix (Applied Biosystems), and variable amounts of water were included in a total of 25 μL reaction volume. The thermocycling conditions followed the initial denaturation conditions recommended by the manufacture: 95 °C for 10 min followed by 35 cycles of 95 °C for 50 s, 56 °C for 40 s, and 72 °C for 60 s for detection and annealing temperature of 64 °C for subtyping. The amplified PCR products were visualized in 1.2% Ethidium bromide gels under UV light. ATCC strains 35150 and 25922 were used as positive and negative controls for *stx* gene detection, respectively.

### 2.4. Screening for Presence of eae and hly in STEC

All STEC were also screened for the intimin gene *eae* and *hly* genes using primers described in Table 1. *E. coli* ATCC 35150 and 25922 were used as positive and negative controls strains for the virulence and genetic markers, respectively.

### 2.5. Determination STEC Serogroups

STEC were also screened with primers specific to select important serogroups (Table 1). Primers targeting all the known *E. coli* serogroups have been recently published for use in molecular epidemiological studies [30]. A literature search was carried out to determine which serogroups had been reported in STEC in goats and sheep in other studies and how frequently they were detected to determine which primers to use for screening the STEC collection in this study. These serogroups included those previously detected in STEC from goats and sheep in previous studies in the US and other countries as well as those detected in disease outbreaks in humans [27,32,33,34,35,36,37,38,39,40,41]. The serogroup primers selected for this study, target genes, and fragment sizes are shown in Table 1. Several PCR reactions utilizing the Amplitaq Gold mastermix, serogroup-specific primers, and annealing temperature based on the individual primer annealing temperatures were carried out to screen the STEC. Amplified PCR products were run on ethidium bromide agarose gels and visualized in a gel imager. Amplified products were confirmed by purification of the PCR products and subsequent sequencing.

### 2.6. Characterization of Phylogenetic Groups

Phylogenetic grouping of *E. coli* can give insight to their pathogenic potential. Currently, eight different phylogroups including seven *E. coli sensu stricto* and one *Escherichia* cryptic clade I are now recognized [31]. In particular, *E. coli* belonging to the phylogroups B2 and D are associated with extra-intestinal infection in humans ([31,42]), while the commensal and intestinal pathogenic strains belong to groups A, B1, and D, as reviewed in [42].The *E. coli* in phylogroups E are related to group D (of which some are 0157:H7), and group F is related to group B2 [39,41,42]. *E. coli* belonging to the latter phylogroups E and F could, thus, have potential pathogenic significance. Each confirmed STEC isolate in this study was subjected to the new Clermont quadruplex PCR, following the previously described protocol and the primers (Table 1) to determine the phylogenetic grouping. The quadruplex PCR detects four sequences: *arpA* (400 bp), *chuA* (288 bp), *yjaA* (211 bp), and *TspE4.C2* (152 bp). On the basis of the results of the quadruplex PCR, subsequent PCRs were carried out to further assign the STEC into the eight currently known phylogroups. The amplified products were electrophoresed in a 2% ethidium bromide agarose gel and visualized under UV light in a gel imager. The isolates were grouped into A, B1, B2, C, D, E, F, or unknown based on the presence or absence of the genes, as described in the protocol.

### 2.7. RAPD Genotyping of STEC

Genetic diversity of STEC was evaluated by Random Amplification of Polymorphic DNA (RAPD) using the M13 RAPD primer. The STEC isolates were grown in Luria broth, and crude DNA lysates were generated by boiling 500 μL of the overnight broth at 100 °C for 10 min. A 1:10 dilution of the boiled lysate was used as the template, as previously described [24]. For the M13 RAPD PCR, the protocol used was that described in [43] Reactions were carried out in a total volume of 25 μL amplification mixtures containing 12.5 μL Dreamtaq 2X master mix, 2.0 μm M13 primer, 2.5 μL crude lysate DNA, and 8 μL molecular grade water. The protocol included an initial denaturation cycle of 95 °C for 3 min, which was followed by 40 cycles of 94 °C for 1 min, 42 °C for 20 s, and 72 °C for 2 min (extension). A final extension cycle was carried out at 72 °C for 10 min. The PCR reaction was carried out in a simpliAmp thermal cycler/USA. The amplified PCR products were run on a 1.5% ethidium bromide agrose gel for 90 min and visualized under UV light. A 1-kb DNA ladder was used as a DNA molecular weight marker. Gels were analyzed manually by evaluating the band sizes generated by the M13 RAPD primer using the 1 Kb ladder. A matrix was generated based on presence (1) or absence (0) of a specific band for each isolate. A similarity index dendrogram was generated using the paired-group UPGMA and Jaccard similarity index using the Past 4.03 software program [44]. Dendrogram visualization, editing, and annotation were carried out using the UPGMA software for generation of Output Dendrogram in Newick Format [45], and the interactive tree of life (iTOL) online tool [46] was used for dendrogram annotation, editing, and visualization.

### 2.8. Statistical Analysis

The STEC data were compiled into proportions using MS Excel descriptive statistics for the various characteristics, then evaluated and compared using the MedCalc comparison of proportions Chi test software for significance differences [47]. This included the proportion of each Shiga toxin type and subtype, the proportion belonging to each serogroup, the age group associated with each serogroup, the proportion belonging to each phylogroup, and the prevalence of the virulence genes in the STEC. Differences were considered significant at *p* < 0.05.

## 3. Results

Four hundred ninety-one (491) STEC from goats were characterized based on their Shiga toxin types, subtypes, serogroups, phylogroups, and presence of the *eae* and *hly* virulence genes. For comparison of the STEC characteristics among age groups represented in the study, the isolates were broadly grouped into goats aged six months and younger (389) and those older than six months (102).

### 3.1. Prevalence of stx1 and stx2 Subtypes in STEC from Goats

**Figure 1:**
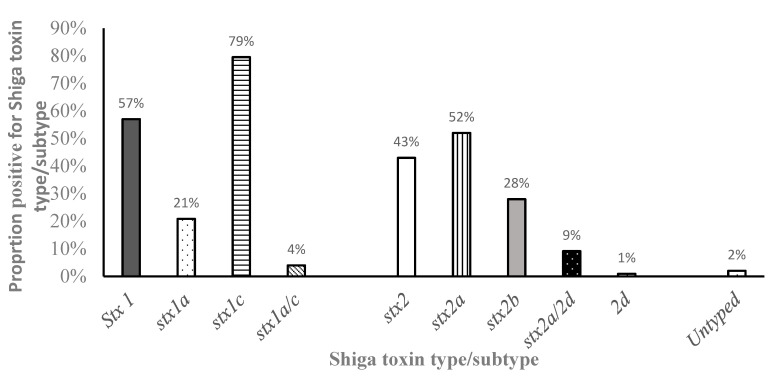
Percentage of *stx1* and *stx2* subtypes in non-O157 (STEC) strains isolated from goats. Note: *stx1a, stx1c, and stx1a/c*—percentage of each subtype out of all STEC-positive for *stx1; stx2a, stx2b, stx2d*, and *stx2a/stx2d*—percentage of each variant/subtype out of all *stx2*-positive STEC.

Among the STEC in this study, the prevalence of isolates with the *stx1* genotype (278 cases, 57%) was significantly higher (*p* < 0.001) than those with the *stx2* genotype (213 cases, 43%) (Figure 1). Among these STEC (491), 95 (19%) had both *stx1* and *stx2* genes There were no observed differences in the distribution of STEC phenotype between age groups. Two Shiga toxin1 subtypes genes were detected, which included *stx1a* and *stx1c*. In comparison, the most common *stx1* subtype (*p* < 0.05) was *stx1*c which was detected in 220 (78%) of all *stx1*-positive STEC. *Stx1a* subtype genes were detected in 58 (21%) of STEC, while 11 (4%) of the STEC isolated had both the *stx1a* and *stx1c* subtype genes. Among the *stx2*-positive STEC, three *stx2* subtype genes were detected, which included *stx2a*, *stx2b*, and *stx2d*. The *stx2a* subtype gene was the predominant *stx2* subtype (*p* < 0.05) being detected in 115 (53%) of STEC, followed by *stx2b* which was detected in 65 (30%), while 20 (9%) had both the *stx2a/2d* subtype genes and two (1%) had only the *stx2d* subtype gene detected (Figure 1).

**Figure 2:**
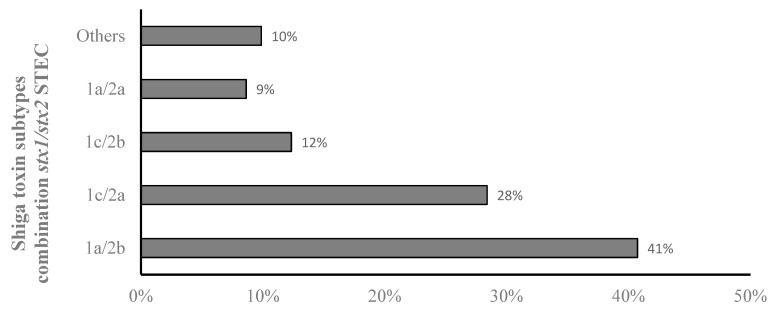
Percentage of *stx* subtypes in STEC harboring *stx1*/*stx2* genotype.

In STEC isolates that harbored both *stx1* and *stx2* genotypes, different combinations of *stx* subtypes were detected. Most of them contained a *stx1a*/*stx2b* genotype (41%), followed by *stx1c*/*stx2a* (28%), *stx1c*/*stx2b* (12%), and *stx1a*/*stx2a* (9%), while all other combinations were detected in 10% of the isolates (Figure 2).

### 3.2. Serogroups of STEC from Goats and Prevalence of Select Virulence Genes

STEC evaluated in this study belonged to ten (11) important serogroups that have been previously detected in goats and some in disease outbreaks in humans (Table 2). Some serogroups were detected at a higher frequency than others and there was an age-based difference in the pattern of distribution of the serogroups among the STEC. Overall, 46% (158) of the 343 serogrouped STEC belonged to O8, 20% (67) to O76, 12% (42) to O91, 5% (17) to O5, and 5% (18) to O26 serogroups. Less than 5% of the STEC belonged to each of the following serogroups: O78, O146, O87, O103, and O121 (Table 2). We observed in this study that STEC belonging to the O8 serogroup were predominantly from goats less than 6 months old (94%, n = 150) compared with adult goats. On the other hand, over 72% (n = 48) of STEC of the O76 serogroup were from goats older than six months of age. All O5 serogroup STEC were isolated from adults, and O26, O103, and 146 STEC were isolated in goat kids 3 months old and younger. STEC belonging to the O91 serogroup were detected across all age groups.

The STEC were screened for two primary virulence genes, *hly* and *eae*. The prevalence of the *hly* virulence gene was significantly higher (*p* < 0.05) than that of *eae* in STEC in this study. Sixty (12%) of the STEC harbored the *eae* gene while two hundred seven (42%) harbored the *hly* gene. Of these STEC, 31 (6.3%) had both the *eae* and *hly* gene detected. The *hly* gene was detected in all serogroups except O78 and O146. In particular, all 42 STEC (100%) belonging to the O91 serogroup harbored the *hly* gene, while it was detected in 80% of those belonging to the O5 and O26 serogroups. In STEC belonging to the serogroups O76 and O8, *hly* was detected in 53% and 33%, respectively. The *eae* gene was detected in O8 (9%), O76 (10%), O91 (5%), O26 (28%), and in two of the three O103 isolates. Isolates with both *eae* and *hly* genes were less than 5% in the O8, O76, and O91 serogroups, 28% in O26 while in the O103 serogroup, two out of the three STEC isolates harbored both genes. The *eae* gene was absent in the O5, O87, O78, O146, and O121 serogroup isolates evaluated in this study (Table 2).

### 3.3. Distribution of Shiga Toxin Types and Subtypes in the Different STEC Serogroups

Overall, the *stx1* Shiga toxin genotype was more commonly detected in all serogroups than the *stx2* and ranged from 30% of isolates in some serogroups to 100% of the isolates in other serogroups (Table 3). On the other hand, *stx2* toxin prevalence ranged from no detection (0%) in some serogroups to 100% in other serogroups. In particular, all STEC belonging to the O91 serogroup (n = 42, 100%) had both the *stx1* and *stx2* Shiga toxins detected. On the other hand, all STEC belonging to the O5 serogroup (n = 15, 100%) and the one isolate belonging to serogroup O121 carried the *stx1* genotype only. The *stx1* genotype was also the most commonly detected (*p* < 0.05) in STEC belonging to the serogroups O76 (n = 67, 87%), O78 (n = 6, 100%), and O103 (n = 3, 100%) compared to *stx2*. Uniquely, in STEC belonging to the O8 serogroup, the *stx2* genotype was detected at significantly higher frequency (*p* < 0.05) than *stx1*. The distribution of the Shiga toxin subtype among the different serogroups was evaluated. In most serogroups with *stx1*, the *stx1*c subtype was the most common (Figure 1). Interestingly, in the O91 serogroup, all STEC (100%) harbored the *stx1a* subtype while the O5, O87, O78, and O146 STEC serogroups had only the *stx1c* subtype detected (Table 3). Both *stx1a* and *stx1c* subtypes were detected in STEC belonging to serogroups O8, O76, O26, and O103 although very few harbored the *stx1a*. Two isolates, one belonging to the O76 and one belonging to the O103 serogroup, had both the *stx1a* and *stx1c* subtype genotype detected. In the eight STEC serogroups with the *stx2* genotype, six (6) of these had only one *stx2* subtype detected (*stx2*a or *stx2*b) (Table 3). Overall, the *stx2*a subtype was the most frequently detected although there were differences among the serogroups (Figure 1). Serogroups with the *stx2*a subtype exclusively included O26, O103, and O146, while those with only the *stx2*b subtype genotype included O91, O87, and O78. In two serogroups (O76 and O8), STEC isolates with either *stx2*a or *stx2*b were detected and in O8 serogroup, isolates with *stx2*d or both *stx2*a/2d genotypes were detected.

### 3.4. Phylogroups of STEC from Goats

**Figure 3:**
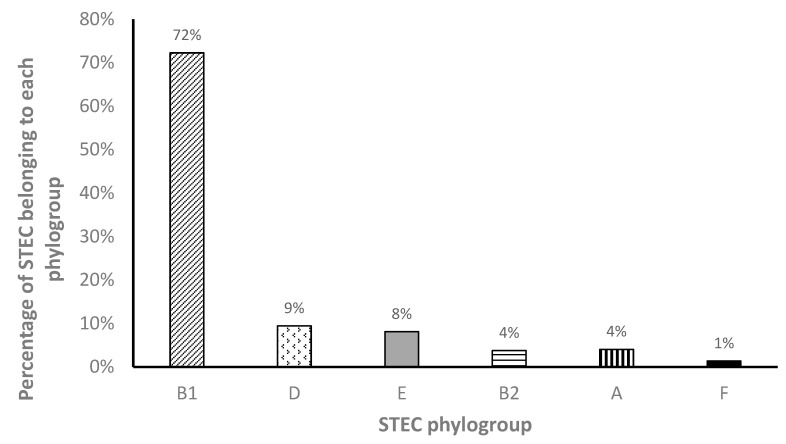
Phylogroups of STEC isolated from goats in the mid-Atlantic US.

Based on the updated Clermont et al. phylogrouping method [31], STEC from goats belonged to six different phylogroups (Figure 3). Over seventy percent (72%) belonged to the phylogroup B1, while 9% and 8% belonged to groups D and E, respectively. Isolates belonging to phylogroups B2 and A were both 4%, while 1% of STEC belonged to phylogroup F.

### 3.5. Genetic Diversity of STEC Strains from Goats as Revealed by M13 RAPD PCR

**Figure 4:**
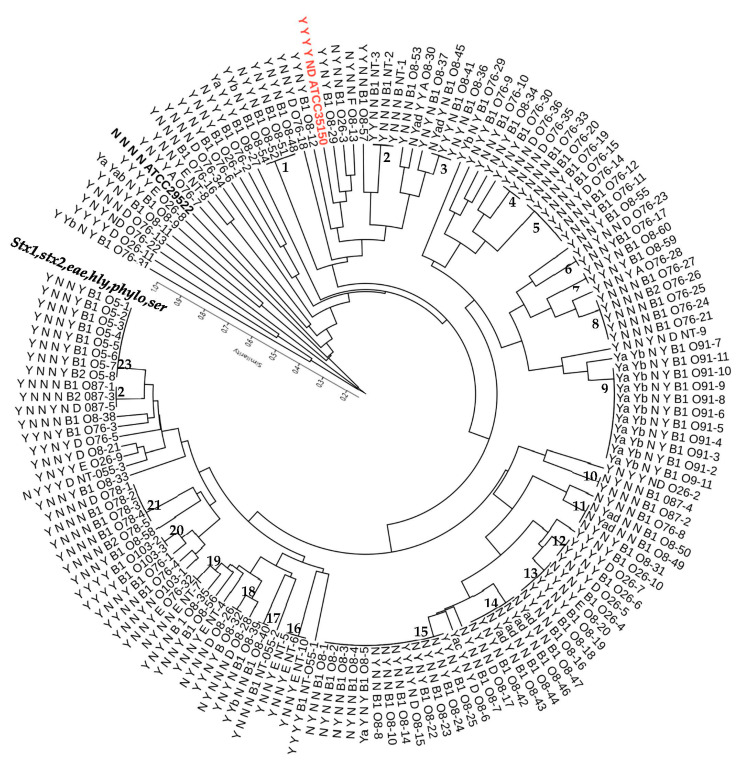
Dendrogram showing overall M13 RAPD genotype clustering of STEC from goats. 1–23 STEC clusters at 95% similarity. Labels in order—{*stx1*; *stx2*; *eae*; *hly* (virulence genes: Y = present, N = none); phylogroups: A, B1, B2, D, E, F, and ND=not determined; serogroups: O5, O8, O26, O76, O78, O87, O91, O103, and NT=non-serogrouped}. *Stx1*: Y = *stx1c* unless indicated. *Stx2*: Y = *stx2a* unless indicated. Bolded black and red-ATCC reference strains (STEC-Red; Non-STEC-black). The highest number of STEC detected in this study belonged to the O8 serogroup. Overall, all the O8 STEC typed in this study shared about 40% genotype patterns based on the M13 RAPD typing, except for two unique isolates (Figure 5). The isolates could be discriminated into six clusters and four unique genotypes at a higher similarity index of 60%. The three clusters at 95% similarity (13, 14, and 15) carried over 40% of the 08 isolates typed. It is worth noting that these three cluster isolates were all shiga toxin 2a-positive but differed in other virulence gene composition and most belonged to the B1 phylogroup. Additionally, four of the six isolates in cluster 14 harbored both *stx2a* and *stx2d* shiga toxin genes. However, no unique virulence gene combination was associated with any of the clusters.

Genetic diversity of goat STEC was identified by M13 primer RAPD PCR using genomic DNA extracted from *E. coli* strains as described in methods above. Two ATCC *E. coli* type strains, STEC (35150-STEC) and a non-STEC (25922), were also included in the RAPD typing. One hundred fifty isolates (152) randomly selected to represent the serogroups as well as shiga toxin genotypes and virulence gene combinations reported in the study were subjected to the M13 RAPD PCR. The RAPD PCR patterns ranged between 3 and 12 bands of fragments that ranged from 300 bp to 3500 bp. Overall, all *E. coli*, including STEC and non-STEC isolates, could be grouped into one main cluster and four other unique genotypes at a 25% similarity index. The STEC evaluated in this study displayed seventy-seven (77) unique RAPD genotypes at a 95% similarity index (Figure 4). At this level, the STEC could be divided into 23 clusters with 2 or more isolates and 44 individual isolate genotypes. These clusters were mostly the same serogroup (21) isolates while some clusters included isolates belonging to two different serogroups (13 and 19).

More than 90% of the goat STEC had a 42% similarity index with ATCC STEC 35150-O157:H7, while similarity was lower with non-STEC strain ATCC 29522, which was approximately 30% (Figure 4). Uniquely, all O5, O91, and O78 STEC had a 100% similarity index (Figure 4) based on M13 RAPD typing. On the other hand, STEC belonging to the other serotypes in this study showed higher RAPD genotype diversity.

Diversity in RAPD genotypes was also detected in the O76 and O26 STEC serogroups whose isolates shared overall 35% within-serogroup genotype pattern (Figure 4). The O76 serogroup isolates were grouped into four clusters at over 95% (clusters 4, 5, 6, and 8) and fifteen single genotypes, but there was no common virulence gene pattern that all the isolates in these clusters shared relative to those not in the clusters. On the other hand, STEC isolates from the O26 serogroup isolates were highly diverse with only three isolates forming one cluster (cluster 12), one isolate in a cluster with O8 (cluster 13), and the rest having unique RAPD genotypes. Similarly, the three isolates in the cluster had no shared unique virulence genotypes different from the other O26 STEC isolates. On the other hand, the three O103 STEC shared over 80% M13 RAPD genotype pattern with two of the isolates showing 100% similarity (cluster 20). The latter two shared the same virulence gene combination and same phylogroup (Figure 4). The five O87 serogroup isolates shared, overall, an 80% similarity M13 RAPD genotype pattern and were discriminated into two clusters at a 100% similarity (clusters 10 and 22). Three of the isolates in the two different clusters shared virulence gene profiles but not phylogroups. Thirteen non-serogrouped (NT) isolates were also included in the M13 RAPD typing PCR (Figure 4). These were discriminated into one major cluster at a 30% similarity and one other single genotype. At a higher similarity level (60%), the isolates were discriminated into two clusters—eight isolates in one and three isolates in another—and two other single genotypes. At a greater than 95% similarity, two clusters each with three isolates (clusters 2 and 16) and seven unique RAPD genotypes could be identified. No unique identifying virulence gene or phylogrouping could be strictly associated with clustering at any of the similarity levels of these isolates.

In some cases, the banding patterns observed from ethidium bromide stained M13 RAPD gels for the STEC in the main clusters at a 95% similarity index displayed unique identifying single dominant band(s). These could be seen in some serogroup clusters or, in some cases, isolates with certain virulence genes. For example, isolates in clusters 1 and 3 shared an approx. 500 bp main band. Conspicuously, all the isolates in these two clusters possessed the *eae* gene. Similarly, *stx2-*positive isolates found in clusters 9, 14, and 15 as well as unique O76 genotypes (Figure 4) had a main band of approx. 1300 bp. This band was present irrespective of the shiga toxin subtype detected (2a, 2ad, or 2b). In contrast, *stx2*-positive cluster 13, belonging to O8 STEC but also possessing *stx1*, had double bands detected instead of a dominant band (data not shown).

## 4. Discussion

Recent studies have shown that the virulence potential of STEC in humans depends on the Shiga toxin subtype carried by the strains. Thus, the Shiga toxin subtypes detected in STEC from potential reservoirs hosts can be used to determine the pathogenic potential of isolates and are useful tools for source tracking in disease epidemiology. In this study, STEC from different age groups of goats from the southeastern US were evaluated for their *stx* gene subtypes. We detected five different Shiga toxin subtypes: *stx1*c, *stx1*a, *stx2*a, *stx2*b, and *stx2*d. Among these, *stx1*c and *stx2*a were the predominant Shiga toxin subtypes detected in STEC from goats from this region. In a previous study from caprine STEC in Iran [48], findings of *stx1* subtypes (*stx1c* and *stx1*a) were reported in goats with *stx1*c being predominant, similar to what was detected in this study. Although the *stx2* subtypes *stx2*a and stx2d detected in our study were also detected in STEC from goats in Iran, no *stx2*b subtype was detected in the latter study. Instead, *stx2*c was also detected which was not detected in our study. In India, *stx1*c subtype was also the predominant Shiga toxin 1, as reported in this study, while *stx2*c and *stx2*d were the only *stx2* subtypes detected [41]. Our findings also agree with studies carried out on STEC from goats and sheep products in Europe, where both *stx1*c and *stx2*b subtypes were detected. No *stx2*a or *stx1*a was detected in meat products from sheep and goats in the latter study unlike in the current study. In a study in China [49] similar to our findings, STEC from goats harbored *stx1*c, *stx1*a, and *stx2*d. However, unlike our study, *stx2*g was also detected in goats in China. These findings, although they overlap on the diversity of Shiga toxin subtypes found in goats, they also indicate that geographical differences exist in the Shiga toxin subtypes found in STEC from goats. Of importance is the predominant Shiga toxin subtype (*stx2*a) detected in STEC from goats from this region in this study. As discussed previously, this subtype has been described as more potent than other *stx2* subtypes in both in vitro and in vivo studies in mice [10]. Morever, the *stx2*a subtype was the predominant subtype in clinical isolates from human disease [7]. This means that healthy goats should be considered potential reservoirs of non-O157 STEC, thus, having high significance in human health.

The STEC evaluated in this study belonged to ten serogroups: O5, O8, O26, O76, O78, O87, O91, O103, O121, and O146. All these have been detected in goats in previous studies in other countries and some in the US [33,36,38,40,41,50]. Eight of these (O5, O8, O26, O76, O91, O103, O121, and O146) are among the top twenty-one most clinically relevant serogroups STEC associated with human infection [51]. Strikingly, three of these—O26, O91, and O103—have been reported at least thirty (30) times in diseased humans, including in the US, while O5, O8, and O146 serogroups have been reported more than 15 times each in diseased human, thus, indicating their significance as public health pathogens [32]. This study further characterized the Shiga toxins types and subtypes associated with STEC serogroups from goats. Some of these serogroups had unique Shiga toxin subtype signatures with all the O91 serogroup having both *stx1* and *stx2* and uniquely having *stx1*a/*stx2*b subtypes. One study in England reported the presence of the O91 serogroup in clinical STEC from diseased humans who were also *stx1*a/*stx2*b-positive [17]. On the other hand, all O5 STEC had the *stx1* toxin and all belonged to the *stx1*c subtype. This information is important for epidemiological disease tracking. Notably, all STEC belonging to most common serogroups associated with human disease evaluated in this study (O26, O103, and O146) carried the *stx2*a subtype which is associated with more severe clinical outcome, further indicating the importance of STEC strains from goats. The majority of O8 STEC serogroups in this study were also *stx2*-positive and harbored the *stx2*a subtype, while those that were *stx1*-positive carried the *stx1*a and *stx1*c genotype. Isolates with similar Shiga toxin subtypes were also detected in cattle in Mexico [52]. The O8 serogroup has been isolated in humans with urinary tract infections [53,54] and bloody diarrhea [55] and in animals—diarrheic lambs, calves [56,57,58], and pigs [59,60] as well as healthy sheep [61]. These results further underscore the importance of STEC from goats.

The presence of other primary virulence genes in STEC from goats evaluated in this study further exemplifies their relevance as public health pathogens. The *eae* gene was detected in STEC belonging to the O8, O26, O76, O91, and O103 serogroups. The *hly* gene was detected in serogroups O5, O8, O26, O76, O87, O91, and O103. Human STEC clinical isolates from Switzerland belonging to the O8, O26, and O103 serogroups were also found to harbor the *eae* and *hly* genes [55]. The STEC in this study belonged to six different phylogroups and there was no specific pattern of distribution of phylogroups among serogroups or Shiga toxin type or subtype status. The majority of STEC in this study belonged to the B1 phylogroup, followed by groups D, E, B2, A, and F. To our knowledge, this is the first report of STEC belonging to phylogroup E from caprine species from this region, a group that includes the *E. coli* strains of the O157: H7 lineage and also phylogroup F based on the new Clermont *E. coli* phylogrouping. Except for phylogroup A that is mostly a commensal, all other phylogroups detected in this study were associated with strains important in causing human disease [62,63]. These findings are similar to studies reported on STEC in caprine species from Iran [48,64,65]; in those studies, phylogroup B1 predominated except in one of the studies [48] where only phylogroups B1 and A were detected. The phylogroups B1 and A were also the most predominant in *E. coli* isolates from respiratory cases in goats in China [66]. Higher percentage of STEC belonging to phylogroup B1 and similar diversity of phylogroups was also detected in wildlife (deer, foxes, and wild boar) in Europe [67] and in calves [68].

We used M13 RAPD genotyping to understand genetic diversity/relatedness of goat STEC belonging to different serogroups and possessing different shiga toxin and two other virulence genes. This technique was successful in amplifying different sized products for all isolates. Our results indicate that STEC from goats are highly diverse, including isolates from the same serogroups in some cases. Although some STEC belonging to some serogroups (O91, O5, O78, O87, and O103) generated highly similar RAPD patterns (>80% similarity), STEC belonging to the O8, O26, and O76 serogroups had higher diversity in the RAPD patterns generated. Nevertheless, the majority of isolates belonging to O8, O26, and the NT groups still had a 60% RAPD genotype similarity index. Within the O8 serogroup, some RAPD patterns were more frequently detected than others and formed major clusters. Although RAPD was able to discriminate STEC based on serogroups in some cases, in serogroups with higher diversity most of the clustering was not based on the presence of a specific shiga toxin, shiga toxin combinations, or similar virulence gene combination. Similar to our findings, [69] did not find clustering of *Escherichia coli* O157:H7 based on virulence genes on RAPD typing. Another study evaluating O157 and non-O157 *E. coli* from cattle and meat also detected intra-serotype M13 RAPD genotype diversity in both groups of strain, even in isolates sharing the same virulence gene combination [70]. In this latter study, similar to our findings, isolates belonging to the O91 serogroup showed similar RAPD pattern. Similarly, another study looking at RAPD patterns of *E. coli* from meat and eggs from different places and presence of shiga toxin detected a high degree of genetic variability, even in shiga toxin-positive *E. coli* from the same species [71]. Similar results indicating within-serogroup RAPD genetic pattern in STEC from healthy goats was reported in India [41].

Our results highlight previously unreported diversity of Shiga toxin subtypes harbored by STEC from goats in the US. The data are important for determining their significance as public health pathogens. In addition, we have elucidated the serogroups and phylogroups to which the STEC belong using the updated Clermont *E. coli* phylogrouping protocol. The M13 RAPD typing of isolates has revealed the genomic diversity and, in some serogroups, the clonality of the STEC isolates from goats. In combination, this information will be useful for disease epidemiology due to *E. coli* surveillance as well as for public education on the risks posed by goats as food and companion animals.

## Figures and Tables

**Figure 5:**
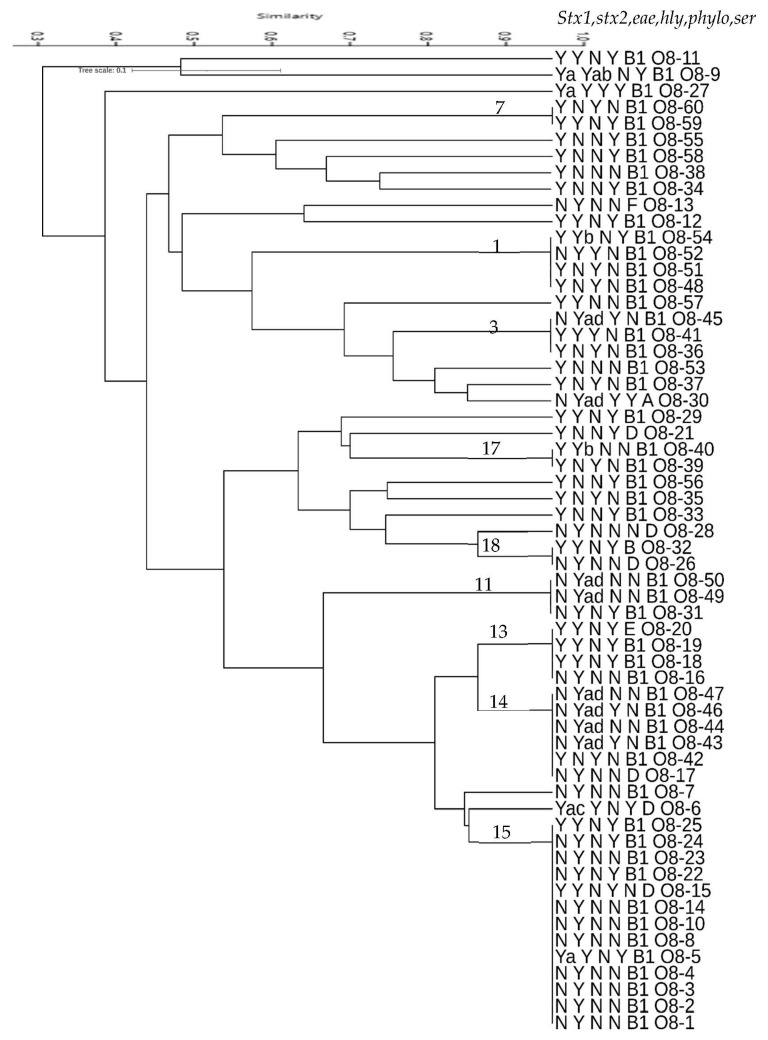
Dendrogram showing overall M13 RAPD genotype clustering of O8 serogroup STEC from goats. 1–18 O8 STEC clusters at 95% similarity. Labels in order—{*stx1; stx2; eae; hly* (virulence genes: Y=present, N=none); phylogroups: A, B1, B2, D, E, F, and ND=not determined; serogroups: O5, O8, O26, O76, O87, O91, O103, and NT=non-serogrouped}. *Stx1*: Y = *stx1c* unless indicated. *Stx2*: Y = *stx2a* unless indicated.

**Table 1:** Primers used in the study. (“—reference same as the one indicated above).

Target Size	Target Gene	Sequence	Primer Name	Ref.
209	*stx1*	GTACGGGGATGCAGATAAATCGC	stx1-det-F1	[7]
		AGCAGTCATTACATAAGAACGYCCACT	stx1-det-R1	
478	*stx1a*	CCTTTCCAGGTACAACAGCGGTT	stx1a-F1	“
		GGAAACTCATCAGATGCCATTCTGG	stx1a-R2	
252	*stx1c*	CCTTTCCTGGTACAACTGCGGTT	stx1c-F1	“
		CAAGTGTTGTACGAAATCCCCTCTGA	stx1c-R1	
203	*stx1d*	CAGTTAATGCGATTGCTAAGGAGTTTACC	stx1d-F1	“
		CTCTTCCTCTGGTTCTAACCCCATGATA	stx1d-R2	
600	*stx2* (all except 2f)	GGCACTGTCTGAAACTGCTCCTGT	F4	“
	*stx2* (all except 2e and 2f)	ATTAAACTGCACTTCAGCAAATCC	R1	
	*stx2 (stx2f)*	CGCTGTCTGAGGCATCTCCGCT	F4-f	
	*stx2* (2e and2f)	TAAACTTCACCTGGGCAAAGCC	R1-e/f	
349	*stx2a*	GCGATACTGRGBACTGTGGCC	stx2a-F2	“
		CCGKCAACCTTCACTGTAAATGTG	stx2a-R3	
347	*stx2a*	GCCACCTTCACTGTGAATGTG	stx2a-R2	“
251	*stx2b*	AAATATGAAGAAGATATTTGTAGCGGC	stx2b-F1	“
		CAGCAAATCCTGAACCTGACG	stx2b-R1	
177	*stxc2*	GAAAGTCACAGTTTTTATATACAACGGGTA	stx2c-F1	“
		CCGGCCACYTTTACTGTGAATGTA	stx2c-R2	
179	*stx2d*	AAARTCACAGTCTTTATATACAACGGGTG	stx2d-F1	“
		TTYCCGGCCACTTTTACTGTG	stx2d-R1	
235	*stx2d-055*	TCAACCGAGCACTTTGCAGTAG	stx2d-O55	“
280	*stx2d*	GCCTGATGCACAGGTACTGGAC	stx2d-R2	“
411	*stx2e*	CGGAGTATCGGGGAGAGGC	stx2e	“
		CTTCCTGACACCTTCACAGTAAAGGT		
424	*stx2f*	TGGGCGTCATTCACTGGTTG	stx2f	“
		TAATGGCCGCCCTGTCTCC		
573	*stx2g*	CACCGGGTAGTTATATTTCTGTGGATATC	stx2g	“
		GATGGCAATTCAGAATAACCGCT		
248	*eaeA*	ATGCTTAGTGCTGGTTTAGG	eaea-a	[28]
		GCCTTCATCATTTCGCTTTC	eaea-b	
569	*hlyA*	AGCTGCAAGTGCGGGTCTG	HlyA-a	“
		TACGGGTTATGCCTGCAAGTTCAC	HlyA-b	
152	*O26wzx*	GCGCTGCAATTGCTTATGTA	Wzx-F	[29]
		TTTCCCCGCAATTTATTCAG	Wzx-R	
527	*O45wzx*	CCGGGTTTCGATTTGTGAAGGTTG	Wzx-F	“
		CACAACAGCCACTACTAGGCAGAA	Wzx-R	
321	*O103wzx*	TTGGAGCGTTAACTGGACCT	Wzx-F	“
		GCTCCCGAGCACGTATAAG	Wzx-R	
925	*O126wzx*	TTAGCTCTCGTAGAGGCTGGTGTT	Wzx-F	“
		ATGTCATTCCTGGGACGCGAATGT	Wzx-R	
640	*O146wzx*	AGGGTGACCATCAACACACTTGGA	wzx-F	“
		AGTTCAATACTGTCGCAGCTCCTC	wzx-R	
566	*O5wzx*	AGGGCAATCTTCCGTAATGA	Og5-PCR_F	[30]
		CCTCTTGGGCTATAAACAACC	Og5-PCR_R	
448	*orf469 (O8)*	CCAGAGGCATAATCAGAAATAACAG	Og8-PCR_F	“
		GCAGAGTTAGTCAACAAAAGGTCAG	Og8-PCR_R	
783	*O6wzy*	GGATGACGATGTGATTTTGGCTAAC	Og6-PCR_F	“
		TCTGGGTTTGCTGTGTATGAGGC	Og6-PCR_R	
207	*O55wzy*	TCCTTATTTGTGTCGGGGG	Og55-PCR_F	“
		CCAGGAAAGCTGCCAATTATC	Og55-PCR_R	
511	*O75wzy*	GAGATATACATGGGGAGGTAGGCT	Og75-PCR_F	“
		ACCCGATAATCATATTCTTCCCAAC	Og75-PCR_R	
457	*O76wzy*	TGGCTTTTATGGCGATATGTG	Og76-PCR_F	“
		TTGTGAGTATAAGCCCCCCAA	Og76-PCR_R	
992	*O78wzx*	GGTATGGGTTTGGTGGTA	Og78-PCR_F	“
		AGAATCACAACTCTCGGCA	Og78-PCR_R	
167	*O87wzy*	GGATGAATGGGGAAAAGCAA	Og87-PCR_F	“
		TCACGCGTAAATCTTCAATCC	Og87-PCR_R	
953	*O91wzy*	GCCTGCGATACCAGTATCCTT	Og91-PCR_F	“
		CCCCCATAATTGGGATCATAT	Og91-PCR_R	
241	*O112wzy*	CGGGTTAACAGCCCATTTTT	Og112ab-PCR_F	“
		CAGCCCCCATTTACCAGTAAT	Og112ab-PCR_R	
782	*O128wzy*	ATGATTTCTTACGGAGTGC	Og128-PCR_F	“
		CTCTAACCTAATCCCTCCC	Og128-PCR_R	
193	*O121wzy*	CAAATGGGCGTTAATACAGCC	Og121-PCR_F	“
		TTCCACCCATCCAACCTCTAA	Og121-PCR_R	
288	*ChuA*	ATGGTACCGGACGAACCAAC	Chua Bf	[31]
		TGCCGCCAGTACCAAAGACA	Chua BR	
211	*yjA*	CAAACGTGAAGTGTCAGGAG	Yja BF	“
		AATGCGTTCCTCAACCTGTG	Yja BR	
152	*TspE4. C2*	CACTATTCGTAAGGTCATCC	TspE4.C2 BF	“
		AGTTTATCGCTGCGGGTCGC	TspE4.C2 BR	
400	*arpA*	AACGCTATTCGCCAGCTTGC	arpA BF	“
		TCTCCCCATACCGTACGCTA	arpA BR	
301	Grp E (*arpA)*	GATTCCATCTTGTCAAAATATGCC	arpA CF	“
		GAAAAGAAAAAGAATTCCCAAGAG	arpA CR	
RAPD	M13	GAGGGTGGCGGTTCT	M13	

**Table 2:** Prevalence of virulence genes in STEC serogroups from goats.

Serogroups (343)	*eae*	*hly*	*eae/hly*
O8 (158)	15 (9.5%)	53 (34%)	3 (1.8%))
O76 (67)	7 (10%)	36 (54%)	3 (4.5%)
O91 (42)	2 (5%)	42 (100%)	2 (5%)
O5 (17)	-	13 (76%)	-
O26 (18)	5 (28%)	14 (78%)	5 (28%)
O78 (6)	-	-	-
O87 (4)	-	1 (25%)	-
O103 (3)	2 (67%)	3 (100%)	2 (67%)
O146 (2)	-	-	-
O121 (1)	-	1 (100%)	-

**Table 3:** Shiga toxin types and subtypes of goat STEC strains belonging to different serogroups.

Serogroups	Percentage	*Stx1*	*Stx2*	*STX1/stx2*	*stx1a*	*stx1c*	*stx1c/stx1a*	*stx2a*	*stx2b*	*stx2d*	*stx2d/2a*
O8 (158)	46%	48 (30%))	107 (68%)	34 (22%)	2 (6%)	46 (96%)	-	75 (70%)	5 (5%)	2 (1.8%)	18 (17%)
O76 (67)	20%	58 (87%)	9 (13%)	7 (10%)	2 (3.4%)	56 (95%)	1 (1.7%)	5 (56%)	4 (44%)	-	-
O91 (42)	12%	42 (100%)	42 (100%)	42 (100%)	42 (100%)	-	-	-	42 (100%)	-	-
O5 (17)	5%	17 (100%)	-	-	-	17 (100%)	-	-	-	-	-
O26 (18)	5%	13 (72%)	12 (67%)	5 (28%)	3 (23%)	10 (77%	-	12 (100%)	-	-	-
O78 (6)	2%	6 (100%)	1 (17%)	1 (17%)	-	6 (100%)	-	-	1 (100%)	-	-
O87 (4)	1%	2 (50%)	2 (50%)	-	-	2 (100%)	-	-	2 (100%)	-	-
O103 (3)	1%	3 (100%)	2 (67%)	2 (67%)	1 (33%)	3 (100%)	1 (33%)	2 (100%)	-	-	-
O146 (2)	1%	1 (50%)	2 (100%)	-	-	1 (100%)	-	2 (100%)	-	-	-
O121 (1)	-	1 (100%)	-	-	-	-	-	-	-	-	-

## Data Availability

All data are available upon request from the corresponding author.
